# Supplementary material for: Content-rich biological network constructed by mining PubMed abstracts
Source: BMC Bioinformatics. 2004 Oct 8;5:147. doi: 10.1186/1471-2105-5-147 (PMC528731; doi:10.1186/1471-2105-5-147)
Supplement: Additional File 2 — The original results of the above study (non-essential files are deleted to keep the file size under the limit set by BMC bioinformatics). [file 1471-2105-5-147-S2.bz2 › chilibotAdditionalFile2/dip10/657ID10353244E11048/html/KPNB1_KPNA2.html]

 


 **KPNB1** and **KPNA2** 
  
Found 172 abstracts in PubMed, retrieved 10.  
 

 What does Google say? 
 PDF only 
| .edu only 

---

**Interactive relationship** (e.g. stimulation, inhibition, etc)

**Neutral relationship**- While performing a yeast two hybrid library screen to uncover novel PP2A interacting proteins, we discovered a specific interaction between a member of the importin beta karyopherin  [ **KPNA2** ]   [ **KPNB1** ]  beta superfamily, importin 9, and the A subunit of PP2A PR65.  Ref: 12670497 Biochem Biophys Res Commun, 2003

**Non-interactive relationship** (e.g. studied together, co-existance, homology, etc.)

- Prospective mimetics with linkers of, 10, or 12 atoms were synthesized and compared in a competition assay for binding to the nuclear import receptor subunit karyopherin  [ **KPNA2** ]   [ **KPNB1** ]  alpha, showing the 10 atom linker to be best and shorter ones ineffective.  Ref: 12841749 Org Lett, 2003
- Treatment with the karyopherin  [ **KPNA2** ]   [ **KPNB1** ]  specific inhibitor leptomycin B was toxic to T. brucei and eliminated the cytoplasmic SL RNA, suggesting that cytoplasmic SL RNA was dependent on the nuclear exporter exportin 1 XPO1.  Ref: 12684371 Eukaryot Cell, 2003
- The NLS of SXR is characterized as the bipartite type, and is recognized by the three molecular species of importin alpha Rch1  [ **KPNA2** ]  PTAC58  [ **KPNA2** ] , NPI1, and Qip1, in the presence of PTAC97  [ **KPNB1** ]  of importin beta to target the nuclear pore.  Ref: 12606758 Mol Pharmacol, 2003
- In the present work, we have characterized the function of the karyopherin  [ **KPNA2** ]   [ **KPNB1** ]  Msn5p in the control of the cell cycle of Saccharomyces cerevisiae.  Ref: 12697814 Mol Cell Biol, 2003
- Second, the import of neoglycoproteins was not affected by microinjection of anti nuclear import factor importin karyopherin  [ **KPNA2** ]   [ **KPNB1** ]  beta antibodies while the NLS dependent transport was completely abolished.  Ref: 12672698 Glycobiology< MedlineTA>Glycobiology, 2003
- In nonpreferred nitrogen or nitrogen starvation, Gln3p is dephosphorylated and imported into the nucleus via karyopherin  [ **KPNA2** ]   [ **KPNB1** ]  alpha Srp1p.  Ref: 12624103 J Biol Chem, 2003
- Kap123p is a yeast beta karyopherin  [ **KPNA2** ]   [ **KPNB1** ]  that imports ribosomal proteins into the nucleus prior to their assembly into preribosomal particles.  Ref: 12612077 Mol Cell Biol, 2003
- Protein cargoes that contain a classical nuclear localization signal NLS are transported into the nucleus through binding to a heterodimeric receptor comprised of importin karyopherin  [ **KPNA2** ]   [ **KPNB1** ]  alpha and beta.  Ref: 12672802 J Biol Chem< MedlineTA>J Biol Chem, 2003
- Most nucleocytoplasmic traffic through the nuclear pore complex is mediated by soluble receptors of the importin exportin or karyopherin  [ **KPNA2** ]   [ **KPNB1** ]  family.  Ref: 12637516 J Biol Chem< MedlineTA>J Biol Chem, 2003
